# The longitudinal effects of the built environment on transportation and recreational walking, and differences by age and sex: systematic review protocol

**DOI:** 10.1186/s13690-023-01194-0

**Published:** 2023-10-17

**Authors:** Tharindu Niwarthana Bandara, Carl Higgs, Belen Zapata-Diomedi, Lucy Gunn, Gavin Turrell, Alysha De Livera

**Affiliations:** 1 RMIT University, Melbourne, Australia; 2 Melbourne School of Population Health, The University of Melbourne, Melbourne, Australia; 3 Department of Mathematics and Statistics, La Trobe University, Melbourne, Australia

**Keywords:** Built environment, Transport walking, Recreational walking, Physical activity, High-income countries, Longitudinal

## Abstract

**Background:**

In high-income countries, the prevalence of physical inactivity and non-communicable diseases is high, and it is now well-established that insufficient physical activity is a risk factor for non-communicable diseases. Walking for recreation and transportation are effective means of improving population levels of physical activity. Research finds that the built environment (BE) can encourage or discourage walking behaviour, and this association varies for different age groups and sexes. This systematic review aims to synthesise longitudinal evidence to better understand how the BE affects recreational and transportation walking for different age groups (above 64 years and 18–64 years) and sexes in high-income countries.

**Method:**

We will use Scopus, PubMed, SPORTDiscus with Full Text (EBSCO), Business Source Complete (EBSCO), Art and Architecture Archive (Proquest), Avery Index to Architectural Periodicals (ProQuest), and Art, Design & Architecture Collection (ProQuest) databases to search for relevant studies. Reviewers will screen the search results according to pre-specified eligibility criteria for study inclusion in the review. Required data for the synthesis will be extracted from the included studies to answer the research questions. Further, the methodological quality of the studies included in this systematic review will be evaluated using an established instrument, and the resulting quality scores will be utilized in sensitivity analysis. The Preferred Reporting Items for Systematic Review and Meta-Analysis (PRISMA) checklist will be followed when reporting the findings.

**Discussion:**

This review will identify BE attributes that are likely to influence transportation and recreational walking for younger and older adults and different sexes in high-income countries. The findings will help policymakers with making decisions around walkable built environments for older and younger adults and different sexes to keep them healthy.

**Trial registration:**

This protocol of the prospective systematic review is developed following PRISMA-P guidelines and is registered on the Prospective Register of Systematic Reviews (PROSPERO) (registration ID CRD42022351919).

## Background

### Rationale

According to the Global Burden of Disease Study [1] and the World Health Organization (WHO)^1^ over 70% of all deaths across the world are caused by non-communicable diseases (NCDs) each year. Of this, 8% relates to insufficient physical activity 2 [2] representing 1.6 million deaths^3^. There is strong evidence that regular physical activity lowers the risk of developing NCDs and premature mortality [2–5]. The WHO recommends at least 150–300 min of moderate-intensity, 75–150 min of vigorous-intensity aerobic physical activity or any equivalent combination of this per week for all adults, to have substantial health benefits [2, 6]. Despite the health benefits, insufficient physical activity is one of the four major modifiable behavioural risk factors contributing to the large health burden from NCDs alongside tobacco use, harmful use of alcohol, and unhealthy diets^4^.

Current literature finds that the population of high-income countries is at high risk of developing and dying from NCDs because of insufficient physical activity. Katzmarzyk et al. [2] found that the percentage of NCD cases attributable to physical inactivity is more than double in high-income countries compared to low-income countries. Further, Guthold et al. [7] found that in 2016 the prevalence of physical inactivity among adults (aged 18 + years) in high-income countries was 36.8% which is more than twice that of low-income countries (16.2%). The authors also found that the prevalence of insufficient physical activity has increased in high-income countries over a 15 year period. This evidence suggests that people in high-income countries are at substantially greater risk of developing NCDs because of physical inactivity. Therefore, understanding the factors that support physical activity is important to mitigate the large global health burden associated with physical inactivity.

Walking is an effective means of reaching recommended physical activity levels that could improve population health. Walking for transportation or recreation can often be incorporated into everyday living because it is easy to do for many people [8, 9], has a low injury risk [10], is cost free and unlike some other activities does not require special equipment or training^5^. An early study by Besser and Dannenberg [11] found that daily walking to and from public transportation solely can help to achieve recommended physical activity levels. Further, numerous studies have found positive health impacts from walking. Results of a review by Boone-Heinonen et al. [12] found that, in general, increase in walking duration, distance, energy expenditure and pace reduces the risk of developing cardiovascular disease, and that this association is stronger for ischemic stroke than coronary heart disease or haemorrhagic stroke. Similarly, a review by Hall et al. [13] concludes from longitudinal evidence that walking an additional 1000 steps per day can reduce the risk of all-cause mortality, and cardiovascular disease morbidity and mortality in adults. Moreover, in their review, Tschentscher et al. [14] conclude Nordic walking (walking with poles) has positive effects on health in terms of resting heart rate, blood pressure, exercise capacity and maximal oxygen consumption. Therefore, walking plays a major role in achieving adults’ required physical activity levels, and so keeping them healthier.

While walking can be encouraged by individual-level programs such as walk-to-work behavioural interventions, the built environment (BE) is viewed by many researchers as a more impactful intervention for promoting walking behaviour [15, 16], since behaviours can be changed more effectively with readily available supportive environments around us than changing minds with individual-level programs [17]. For example, Audrey et al. [18] concluded that having BE attributes such as whether or not there is a carpark, availability of public transport and distance to workplace can even influence the effectiveness of the individual-level programs. The BE effects on walking have been widely explored by researchers for many years and results indicate that BE attributes influence walking behaviour [15, 19]. Certain BE attributes are differently associated with walking which can be undertaken for transportation or recreational purposes. A review by Cerin et al. [20] shows that residential density, walkability, street connectivity, overall access to destinations, land use mix and pedestrian-friendly features are positively related to transportation walking. In another review, Van Cauwenberg et al. [21] found that walkability, land-use mix-access and aesthetically pleasing scenery were positively associated with recreational (leisure-time) walking. Furthermore, several studies have found that some BE characteristics (e.g., walkability and land use mix) in general are more likely to be associated with transportation walking than recreational walking [22, 23]. In addition, identification of specific BE attributes that determine specific behaviour (walking for transportation, walking for recreation) has been highlighted as vital to improve the predictive capacity of ecological models [24]. Motivated by these factors, this review intends to evaluate BE effect on walking for transportation and recreation.

The literature has highlighted the importance of separately assessing BE effect on walking for different sexes and older and younger adults. However, little is known about which specific BE attributes encourage transportation walking and recreational walking for different groups of people (males, females, younger adults, and older adults). Few studies have explored the role of these demographics in the association between BE attributes and walking behaviour. For example, the distance older adults travel from their homes decreases with age [20], and also their physical inactivity increases significantly with increasing age [25]. Therefore, BE attributes that motivate, or limit walking vary with age. Moreover, Lee et al. [26] concluded that older adults were motivated to walk by the presence of even and smooth walking surfaces and benches, and younger adults were strongly motivated by proximity to recreational facilities for walking. In addition, the authors also observed significant age differences for safety-related barriers, fear of injury, and traffic safety concerns. Therefore, reporting BE effects for all adults (over 18 years) collectively, instead of separately evaluating the effects for older adults (over 65 years) and younger adults (aged between 18 and 65 years), may lead to confounded results. Similarly, men and women tend to use their neighbourhoods in different ways at different ages. Cerin et al. [20] reported that sex has a moderating effect on associations between BE and transportation walking and cycling. Using mostly cross-sectional studies, the authors also reported that they observed a complex age-sex interaction effect. Another cross-sectional study reported that neighbourhood-level factors influence recreational walking differently for men and women [27], indicating increased environmental sensitivity among women [27, 28]. Furthermore, Tcymbal et al. [29] conducted a review of the BE effect on physical activity using longitudinal studies taking sex into account, although the authors did not focus on different age groups. According to the authors, the availability of public transport, safe cycling lanes, housing density, and distance to daily destinations were more relevant for women’s physical activity than for men. Whilst street network characteristics and road environments, such as intersection connectivity, local road density, and the presence of dead-end roads were found to be more relevant for men’s physical activity than for women. Hence, motivated by the above evidence, this review will seek to synthesize the findings on BE association with transportation and recreational walking for different sexes and older and younger adults with a focus on longitudinal studies, since longitudinal studies allow exploration of prospective effects. Studies from high-income countries will be used for this synthesis, and high-income countries will be identified according to The World Bank [30] classification.

### Objective

Despite a large amount of empirical evidence and systematic reviews of the BE effects on walking, less is known about how the BE effects on recreational and transportation walking differ by age and sex in high-income countries as identified by The World Bank [30]. This systematic review aims to address this knowledge gap by answering the question of whether the longitudinal effects of the BE on recreational and transportation walking differ by older (above 64 years) and younger (18–64 years) adults and by sex in high-income countries.

## Methods

This systematic review protocol was developed according to the preferred reporting items for systematic review and meta-analysis protocols (PRISMA-P) checklist.

### Eligibility criteria

1. Published and peer-reviewed empirical studies in the English language.
2. Studies must have been conducted in high-income countries (according to The World Bank [30] classification).
3. Empirical results based on longitudinal, repeated cross-sectional, natural-experiment and quasi-experiment study designs (cross-sectional designs are excluded).
4. Must have studied and estimated the BE effects on transportation or recreational walking.
5. Must have studied the effects either on working-aged adults (aged between 18 and 64 years) or/and older adults separately (older than 64 years). Children or adolescents are excluded.
6. BE attributes must be objectively measured.
7. Studies must have been conducted in urban areas (functional urban areas will be identified using the European Commission [31] classification).
8. Must have studied the effects on the general population and not on specific population subgroups (other than age and/or sex) such as people with specific medical conditions.

### Information sources

We will search articles on the BE and walking behaviour in health, behavioural sciences, sport, environmental social sciences, transportation, urban design, physical activity, and multi-disciplinary databases, based on guidance from a team of researchers and librarians to sufficiently cover the topic. The selected databases are: Scopus, PubMed, SPORTDiscus with Full Text (EBSCO), Business Source Complete (EBSCO), Art and Architecture Archive (Proquest), Avery Index to Architectural Periodicals (ProQuest) and Art, Design & Architecture Collection (ProQuest).

### Search strategy

The search will be conducted using the search terms listed in Table 1 in the Appendix, which is divided into four sections for BE, walking, purpose of activity (transportation or recreational) and study design. The search terms for each section were identified by referring to those used in other relevant reviews [20, 21, 32, 33]. The terms listed in each of these four sections will be searched in titles and abstracts of studies using OR Boolean operator, and then the results of these four searches will be combined using AND Boolean operator. An example of the search queries used for the Scopus database registry is given in Table 2 in the Appendix. Finally, systematic reviews are excluded, and the studies will be filtered to ensure they are peer-reviewed and published in the English language. The search was carried out for titles and abstracts of articles published until 30th June 2022 and the resulted number of studies is given in Table 3 in the Appendix.

### Study records

Once the search is completed duplicate studies will be removed using the EndNote reference manager, and titles and abstracts screened based on the inclusion criteria following the method described by Bramer et al. [34]. Lastly, full texts will be reviewed. Two reviewers (TB and CH) will independently screen all studies identified in the search and a third reviewer will decide on discrepancies. Once the studies are selected, the reviewers will be given a form (Table 4 in the Appendix) to extract the required data. Reviewers will independently record the data from all selected studies and will confirm discrepancies by discussion until consensus is reached.

### Data items

We will extract the type of BE attributes or interventions as exposures, exposure measurement details, outcomes (walking for transportation and/or recreation), measurement details of the outcome, covariates used, statistical approach, findings (estimates of the associations with confidence intervals, significance status and sample sizes), study characteristics (authors, year, study design, follow-up period, location of the study etc.) and participants’ characteristics (age, sex, etc.).

### Outcomes and prioritization

The primary outcomes of this systematic review are walking for recreation and walking for transportation.

### Risk of bias in individual studies (methodological quality assessment)

The quality of the included articles will be assessed using 14 criteria mirroring those used in a previous relatable systematic review [32], these criteria were developed based on those used by Cerin et al. [20]. Items and the score allocations of the quality criteria tool are summarized in Table 5 in the Appendix. As described by Chandrabose et al., a score of 1 or 0 will be assigned for each assessment item depending on whether each item meets the quality criteria or not respectively. A score of 0.5 will be assigned if an item is at an acceptable level of its criteria. A score of 0 or 1/3 will be assigned for items 6, 7 and 11 to avoid overstating the importance of statistical aspects of the articles. An additional score will be given for sample size for each study as described by Chandrabose et al., [32] and Cerin et al. [20] in their reviews. The scores for sample sizes are as follows: 0.25 for sample size ≤ 100; 0.5 for sample size 101–300; 1 for sample size 301–500; 1.25 for sample size 501–1000; 1.5 for sample size 1001–2500; and 1.75 for sample size ≥ 2500. The sum of the quality assessment score and sample size score will be assigned to each included study to assess the strength of evidence towards the research questions. The total quality scores will be used as weights when synthesising the data as described in the Data synthesis section.

### Data synthesis

We will summarize the reported evidence to answer our research questions. All reported unique associations between BE attributes and walking will be summarized as number of positive (i.e., BE attributes that support walking, for example, high walkability that encourages walking) and negative (i.e., BE attributes that discourage walking) associations found into Table 6 in the Appendix. In the case of finding non-linear relationships, we will report these findings in the tables and discuss them in detail in the discussion.

The effects of the BE on transportation and recreational walking will be summarized separately for males and females aged 18–64 years, as well as for older adults aged 65 years and older. For example, if an included study looked at the effects only for males aged 18–45 years, this evidence will be used to synthesise evidence for younger male adults. Evidence from studies that have not been separated by sex will be considered to assess the effects for younger and older adults, to compare the effects between younger and older adults irrespective of sex. As a sensitivity analysis, we will exclude evidence from low-quality studies.

### Meta-bias(es)

When a protocol is available for a study, we will compare whether all the planned outcomes in the protocol are reported in the published study to identify the existence of selective outcome reporting bias. This bias occurs when study outcomes are reported according to the researchers’ choice of significance, magnitude or direction of the outcomes [35], instead of reporting all intended study outcomes.

### Confidence in cumulative evidence

Quality of the evidence (also known as certainty of the evidence) for all outcomes will be graded using the Grading of Recommendations Assessment, Development and Evaluation (GRADE) tool [36]. The quality of the evidence will be graded into four levels: very low (the true effect is probably noticeably different to the estimated effect), low (the true effect might be noticeably different to the estimated effect), moderate (the true effect is probably close to the estimated effect) and high (the true effect is very likely to be the estimated effect). As described by GRADE, the rating for the quality of outcomes for randomized controlled trials is set as high-quality, whilst observational studies are set as low-quality. Then the quality of outcomes is rated down for five categories; (i) risk of bias – limitations in the study design, (ii) inconsistency of results – heterogeneity or variability in results (treatment effect) across studies, (iii) indirectness of evidence – not directly comparing the intervention of interest for the population of interest, (iv) imprecision – having wide 95% confidence interval (CI) around the estimate of the effect when relatively small sample size is used, (v) publication bias – bias due to selective publication of studies. Likewise, the quality of outcomes is rated up for three categories; (i) large magnitude of effect, (ii) exposure-response gradient - increasing level of exposure is associated with either an increasing or a decreasing of outcome, (iii) residual confounding – adjusting for prognostic factors that may relate to the outcome of interest.

The causal effect of an intervention can only be inferred through a randomised allocation of exposure [37], therefore randomised studies are rated as high-quality studies. However, it is likely that a greater number of observational BE-behaviour studies will be included in our review due to the ethical and implementation challenges of randomized controlled trials for evaluating the behavioural impact of BE interventions. Natural experiments, which are observational studies may support stronger causal inferences. They are increasingly common in public health and their strength of evidence is higher than in other observational studies [37]. Also, a conceptual article from the GRADE Public Health Group identifies this challenge and suggests that natural experiments (i.e., interrupted time series or regression discontinuity studies) should be rated as moderate quality [38]. Therefore, the quality of outcomes from natural experiment studies will be rated as moderate quality.

## Discussion

The prevalence of physical inactivity and non-communicable diseases is high in high-income countries. Walking is an effective way to achieve recommended levels of physical activity and promote healthy living. The BE is one of the factors that determine walking behaviour, and this systematic review aims to identify the BE attributes that influence transportation and recreational walking in the literature. This association will be separately assessed for younger adults and older adults, as well as for males and females, as literature suggests that this association differs among these groups.

This evidence synthesis considers peer-reviewed published longitudinal studies. We will provide a series of recommendations based on the resulting evidence synthesis and will disseminate our findings through existing professional networks and platforms such as LinkedIn and Twitter. The findings of this review will assist policymakers in making decisions regarding the provision of walkable neighbourhoods for residents in high-income countries, thereby promoting healthy living.

## Data Availability

Not applicable.

## Appendix

**Table 1:** Database search terms for systematic review of longitudinal built environment attributes associated with transportation and recreational walking

**Built environment**
**General**
**“built environment*” OR neighbourhood* OR neighborhood* OR “physical** environment*” OR geograph* OR environment* OR “urban environment*” OR “objective environment*” OR spatial OR “pedestrian-friendly” OR “suburban environment*”
**Built Environment characteristics**
sprawl OR “street connectivity” OR “land use” OR walkab* OR sidewalk* OR footpath* OR “urban design*” OR “urban form” OR “community design*” OR “spatial cluster*” OR “bike way*” OR “bike path*” OR “bike lane*” OR “foot path*” OR “side walk*” OR “cycle path*” OR “bicycle path*” OR “bicycle lane*”
**Conditions**
speed limit* OR traffic OR low traffic
**Densities and Destinations**
destination* OR “residential density” OR “population density” OR “employment density” OR “retail density” OR proximity OR accessibility OR “recreation* resource*” OR “recreation* facilit*” OR “recreation* opportunit*”
**Open space**
park OR parks OR “open space*” OR garden* OR green*
**Walking**
walk* OR strolling OR “active transport*” OR “ecological commut*” OR “ecological transport*” OR “non-auto*” OR “non-motorise*” OR “non-motorize*” OR travel OR “active travel*” OR ambulation
**Physical activity**
“physical activity” OR exercise OR inactive* OR “physically active*”
**Purpose of walking**
transport* OR recreation* OR leisure* OR “activity purpose” OR “travel purpose” OR utilitarian
**Study design**
longitudinal* OR prospectiv* OR cohort OR “follow-up” OR “follow up” OR relocat* OR stay* OR move*
experiment* OR quasi OR “quasi-experiment*” OR “before-after” OR “time series” OR “prospective stud*” OR “natural experiment*”
“repeated cross-section*”
AND NOT “systematic review*”

**Table 2:** An example of search queries used in Scopus database registry

**Query used for built environment**
TITLE-ABS ( “built environment*” OR neighbourhood* OR neighborhood* OR “physical environment*” OR geograph* OR environment* OR “urban environment*” OR “objective environment*” OR spatial OR pedestrian-friendly OR “suburban environment” OR sprawl OR “street connectivity” OR “land use” OR walkab* OR sidewalk* OR footpath* OR “urban design*” OR “urban form” OR “community design*” OR “spatial cluster*” OR “bike way*” OR “bike path*” OR “bike lane*” OR “foot path*” OR “side walk*” OR “cycle path*” OR “bicycle path*” OR “bicycle lane*” OR “speed limit*” OR traffic OR “low traffic” OR destination* OR “residential density” OR “population density” OR “employment density” OR “retail density” OR proximity OR accessibility OR “recreation* resource*” OR “recreation* facilit*” OR “recreation* opportunit*” OR park OR parks OR “open space*” OR garden* OR green* )
**Query used for walking**
TITLE-ABS ( walk* OR strolling OR “active transport*” OR “ecological commut*” OR “ecological transport*” OR non-auto* OR non-motorise* OR non-motorize* OR travel OR “active travel*” OR “physical activity” OR exercise OR inactive* OR “physically active*” OR ambulation )
**Query used for study design**
TITLE-ABS ( longitudinal* OR prospectiv* OR cohort OR follow-up OR “follow up” OR relocat* OR stay* OR mover* OR experiment* OR quasi OR quasi-experiment* OR “before-after” OR “time series” OR “prospective stud*” OR “natural experiment*” OR “repeated cross-section*” )
**Query used for purpose of walking**
TITLE-ABS ( transport* OR recreation* OR leisure* OR “activity purpose” OR “travel purpose” OR utilitarian )

**Table 3:** Article search results by database registry

Database registry [databases]	Number of results
Scopus	7073
PubMed	2075
EBSCO [SPORTDiscus with Full Text, Business Source Complete]	409
ProQuest [Art and Architecture Archive, Avery Index to Architectural Periodicals, Art, Design & Architecture Collection]	20

**Table 4:** Systematic review data to be extracted from included studies

General	Study ID
	Author, year
	Country
	Sample size
	Sex
	Age group
	Socio-economic groups
For quality assessment	Study design; Data source [Baseline response rate; Retention at last follow-up; Multisite or not; Recruitment is done by stratified environmental attributes or not]
	Mean follow-up duration (Data collection period)
	Number of data collection time-points (Multiple time-points exposure measures or not/ Temporal match of exposure-outcome)
	Residential relocation status of participants
	Walking Outcome Variable(s) (Measurement type) Measurement method
	Built Environment Exposure Variable(s) (Measurement method)
	Geographic Scale
Analysis and findings	Findings
	Statistical Method(s)
	Confounders
	Moderators

**Table 5:** Systematic review quality assessment items and scoring criteria

Item No.	Quality assessment item	Score allocation
[1]	Sample representativeness of the population	Response rate ≥ 60% or sample shown to be representative of the population • Yes = 1.0 • No = 0.0
[2]	Study design	• Natural experimental = 1.0 • Observational = 0.5
[3]	Exposure variability (study areas selected to maximize the variability in the exposure variables)	Recruitment stratified by key built environmental attributes • Yes = 1.0 • No = 0.0
[4]	Adjustment for individual socio-demographic covariates (age, sex, education or similar)	• Yes = 1.0 • No = 0.0
[5]	Adjustment for residential self-selection	• Directly adjusted = 1.0 • Accounted by alternative approaches = 0.5 • Not accounted = 0.0
[6]	Analytical approach accounted for area-level clustering (if appropriate)	• Yes = 1/3 • No = 0.0
[7]	Appropriate presentation of analysis results (i.e., formal testing of moderators, if applicable; presentation of point estimates and 95% confidence intervals, standard errors and/or p-values)	• Yes = 1/3 • No = 0.0
[8]	Follow-up duration	• 5 + years = 1.0 • 2 to 5 years = 0.5 • < 2 years = 0.0
[9]	Number of data collection time points	• 3 + waves = 1.0 • 2 waves & health registries = 0.5
[10]	Participant retention rate	• Retention rate ≥ 80% or drop-outs are comparable with follow-up participants = 1.0 • 50% ≤ retention rate < 80% = 0.5 • Retention rate < 50% or drop-outs are non-comparable with follow-up participants = 0.0
[11]	Appropriate longitudinal data analysis	• Yes = 1/3 • No = 0.0
[12]	Measurement of built environment attributes (appropriate geographical unit and size to capture participants’ neighbourhood for objective measures)	Objectively measured built environment: • Street-network buffer = 1.0 • Straight-line (crow-fly) buffer or smaller administrative units = 0.5 • Larger administrative units = 0.0 Perceived built environment: Measured using validated survey instruments? • Yes = 1.0 • No = 0.0
[13]	Measurement of outcome (objectively measured vs. self-reported)	• Objective = 1.0 • Self-reported = 0.5
[14]	Temporal match of exposure and outcome measures	• Multiple time-points exposure measures concurrent with outcome measures = 1.0 • Single time-point exposure measure (within the follow-up period) = 0.5 • Single time-point exposure measure (outside of the follow-up period) = 0.0

**Table 6:** Unpopulated systematic review results summary table for transportation and recreational walking by age and sex in high income countries

BE attributes	**Walking**										Total walking
	Transport Walking				Total Transport walking^a^	Recreational Walking				Total Recreational walking^a^	
	Younger adults		Older adults			Younger adults		Older adults			
	Male	Female	Male	Female		Male	Female	Male	Female		

^a^ Total transport walking includes walking for transportation by both younger and older adults irrespective of sex, and total recreational walking includes walking for leisure by both younger and older adults irrespective of sex

## Abbreviations

PRISMA: Preferred Reporting Items for Systematic Review and Meta-Analysis
PROSPERO: Prospective Register of Systematic Reviews
BE: Built Environment
WHO: The World Health Organization
NCDs: Non-Communicable Diseases
GRADE: Grading of Recommendations Assessment, Development and Evaluation

## Footnotes

1 World Health Organization – Non-communicable diseases (https://www.who.int/news-room/fact-sheets/detail/noncommunicable-diseases).

2 BMJ – (https://www.bmj.com/company/newsroom/physical-inactivity-is-responsible-for-up-to-8-of-non-communicable-diseases-and-deaths-worldwide/#:~:text=With%20physical%20inactivity%20increasing%20across,disease%20associated%20with%20physical%20inactivity.)

3 World Health Organization - Noncommunicable diseases (https://www.who.int/news-room/fact-sheets/detail/noncommunicable-diseases).

4 World Health Organization - Noncommunicable diseases (https://www.who.int/news-room/fact-sheets/detail/noncommunicable-diseases).

5 Better Health Channel – Walking for good health (https://www.betterhealth.vic.gov.au/health/healthyliving/walking-for-good-health).
